# Main Determinants of Ossification in Humerus Bone and Coracoid Process among Iranian Neonates

**Published:** 2017-11

**Authors:** Azadeh MEMARIAN, Shahrokh MEHRPISHEH, Elham ZAREI, Farrokh TAPHTACHI, Leila ABDOLKARIMI

**Affiliations:** 1. Dept. of Forensic Medicine, School of Medicine, Iran University of Medical Sciences, Tehran, Iran; 2. Dept. of Neonatology, School of Medicine, Qazvin University of Medical Sciences, Qazvin, Iran; 3. Dept. of Radiology, School of Medicine, Iran University of Medical Sciences, Tehran, Iran

## Dear Editor-in-Chief

About ossification of the coracoid process, this part is naturally cartilaginous at birth, while ossification takes place in the middle of the coracoid process from fifteenth to eighteenth months after birth (1). More importantly, not only the ossification in humerus and coracoid is processed on a normal embryological pattern, but these processes can be also affected by some neonatal and maternal factors such as gestational age and birth weight (2–4). Thus, the processes of ossification and formation in these two bones should be assessed by considering other maternal and neonatal indicators. The present study aimed to first determine the condition of ossification in humerus and coracoid process and then to determine main indicators of these processes among a sample of Iranian newborns. We performed a cross-sectional study on 150 Iranian newborns (less than 28 d) hospitalized at Rasoole-Akram Hospital in Tehran, Iran during 2014. The baseline information, as well as laboratory parameters such as serum levels of calcium and phosphorus, was collected by reviewing the hospital-recorded files and recorded at the study checklists. The appearance of the epiphyseal centers for humerus and coracoid process were tested by radiographic assessment of these bones. In the present study, by radiography as an available tool for assessing bone ossification in neonates, we assessed the prevalence rate of ossification in two bones including humerus and coracoid process in a sample of Iranian neonates. In total, humerus epiphysis was revealed in 16% of neonates, while coracoid ossification was ossified in 8% of them. The main determinants of humerus ossification included height of neonates, while the main correlates of coracoid ossification were underlying disorders, normal vaginal delivery, and serum calcium level. In this regard, epiphysis ossification was directly associated with gestational age that between 66.7% and 79.2% of ossification appeared in term neonates that are comparable with some previous reports.

In total, along with gestational age as main determinant for ossification of two bones including humerus and coracoid, ossification in these bones can be associated with body weight, mode of delivery and even some underlying disorders affecting bone formation. In addition, coracoid epiphysis was appeared in 8% (66.7% in term infants and 33.3% in preterm infants). However, the ossification of these bones is independent of gender of neonates. In addition, according to our result, the rate of ossification in humerus is about two times of coracoid during neonates, whereas it may not be earlier in humerus than in coracoid. The ossification of coracoid epiphysis was related to the presence of icterus, IUGR or anomaly as well as to normal vaginal delivery, lower serum calcium level, and higher mean height. In overall, the latter finding should be more assessed in various populations.
